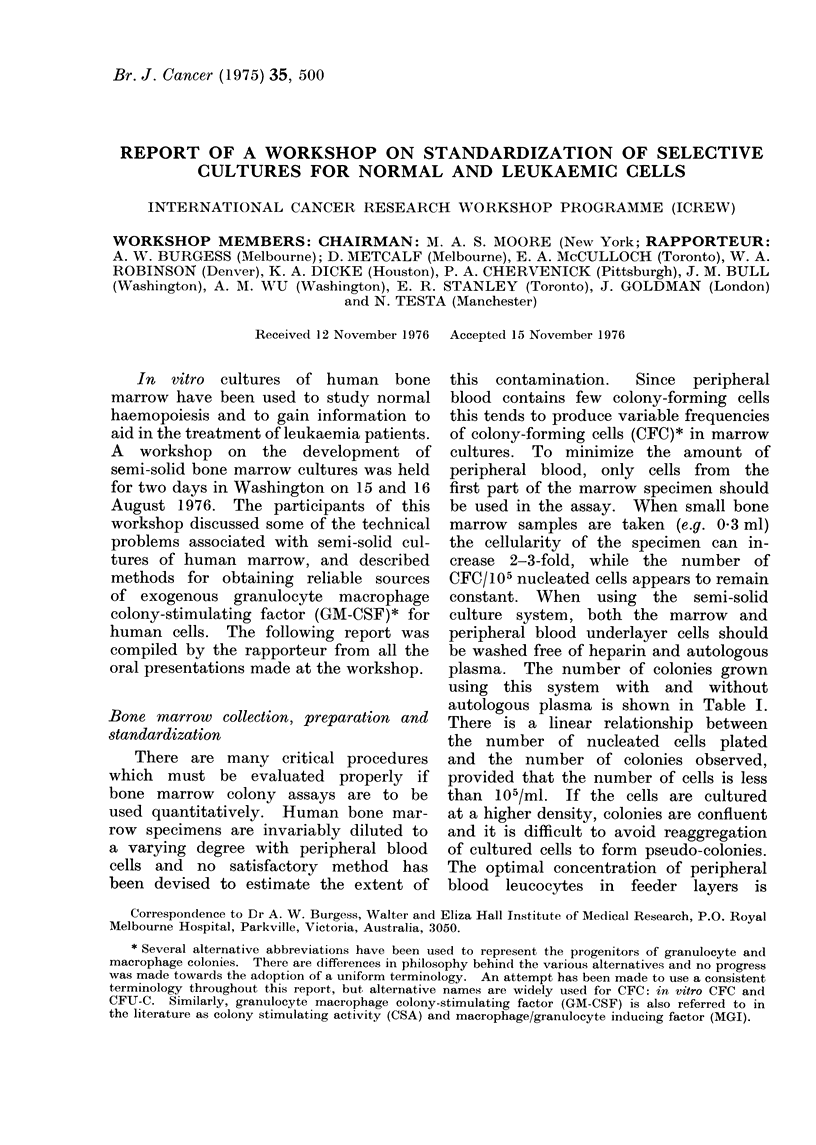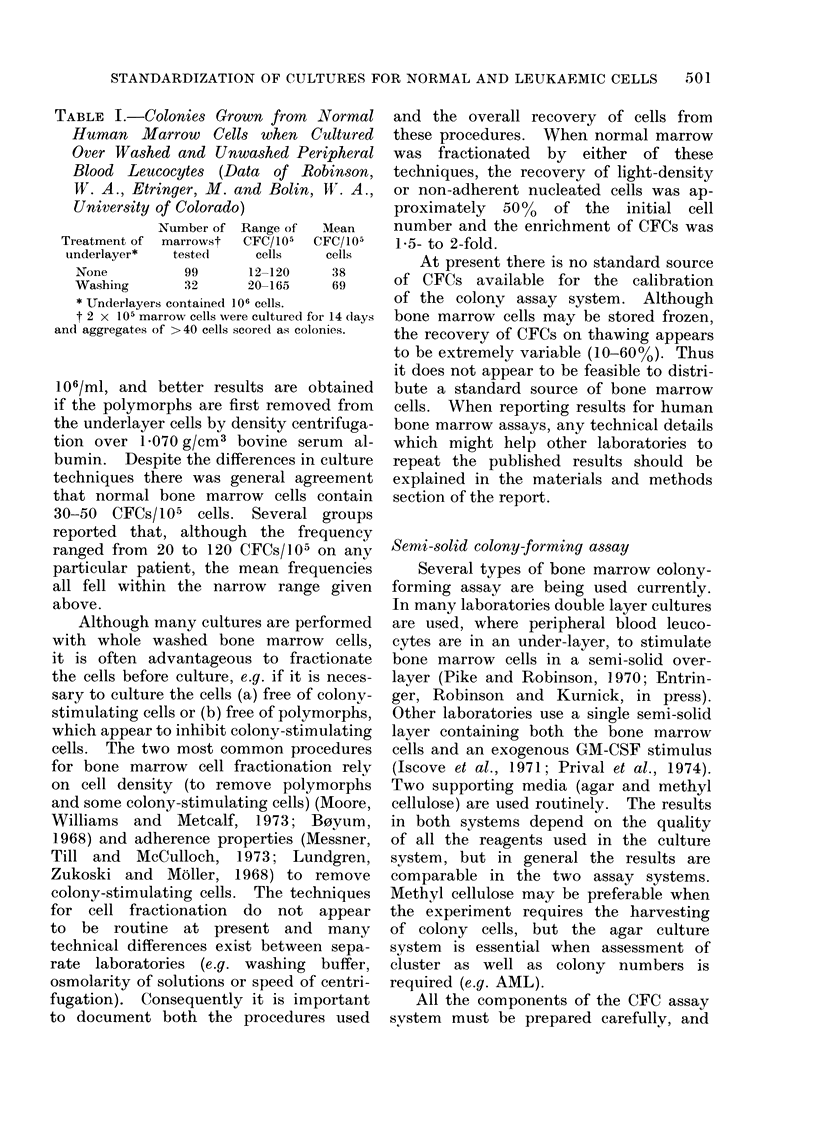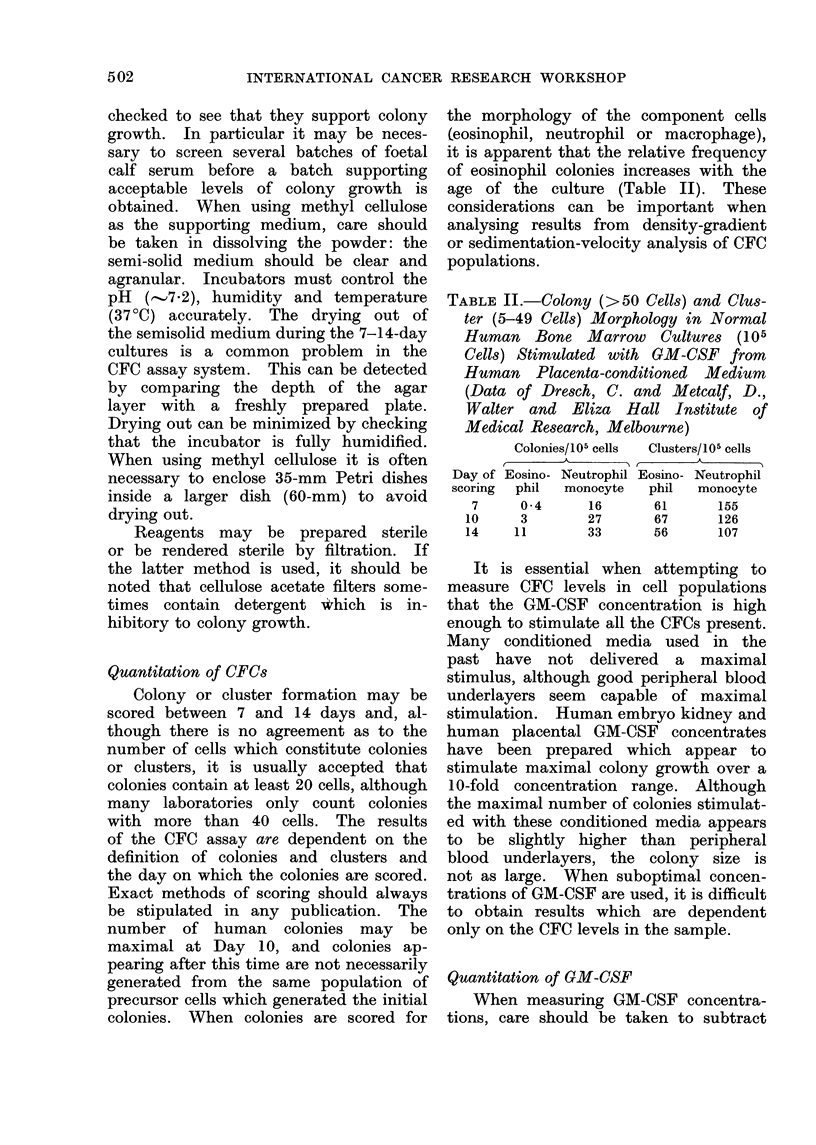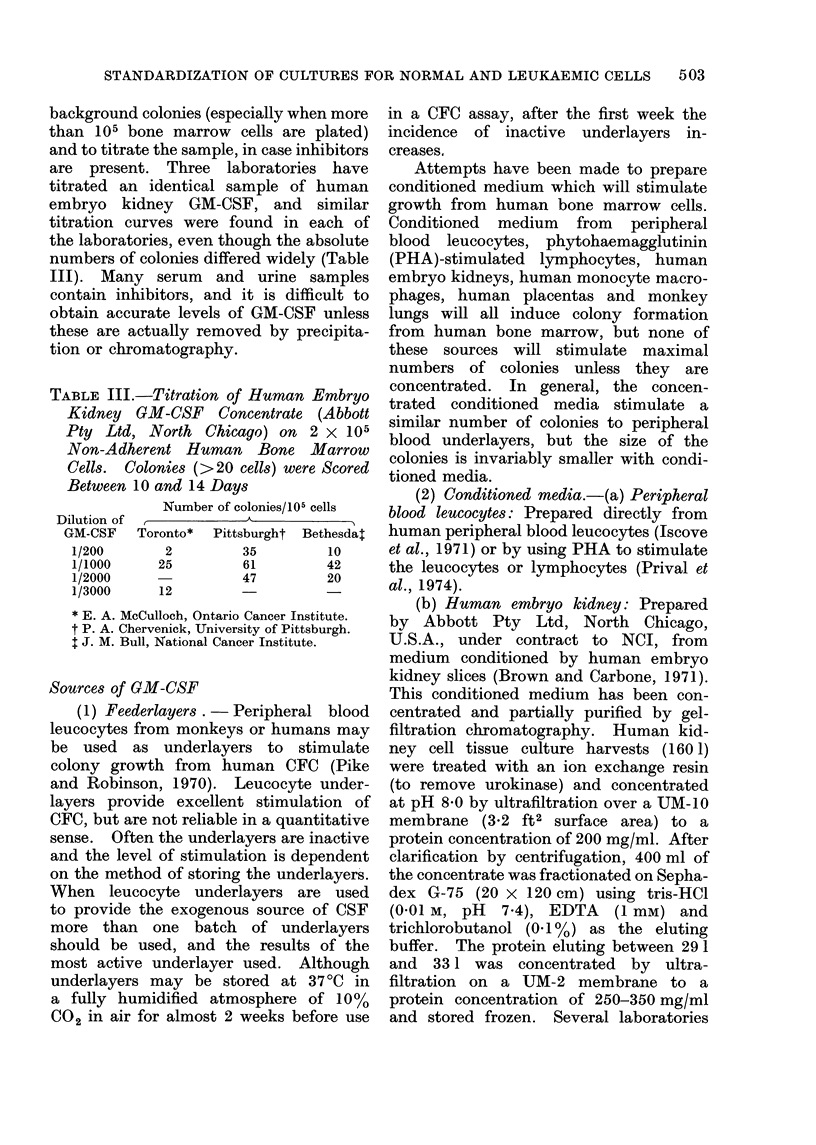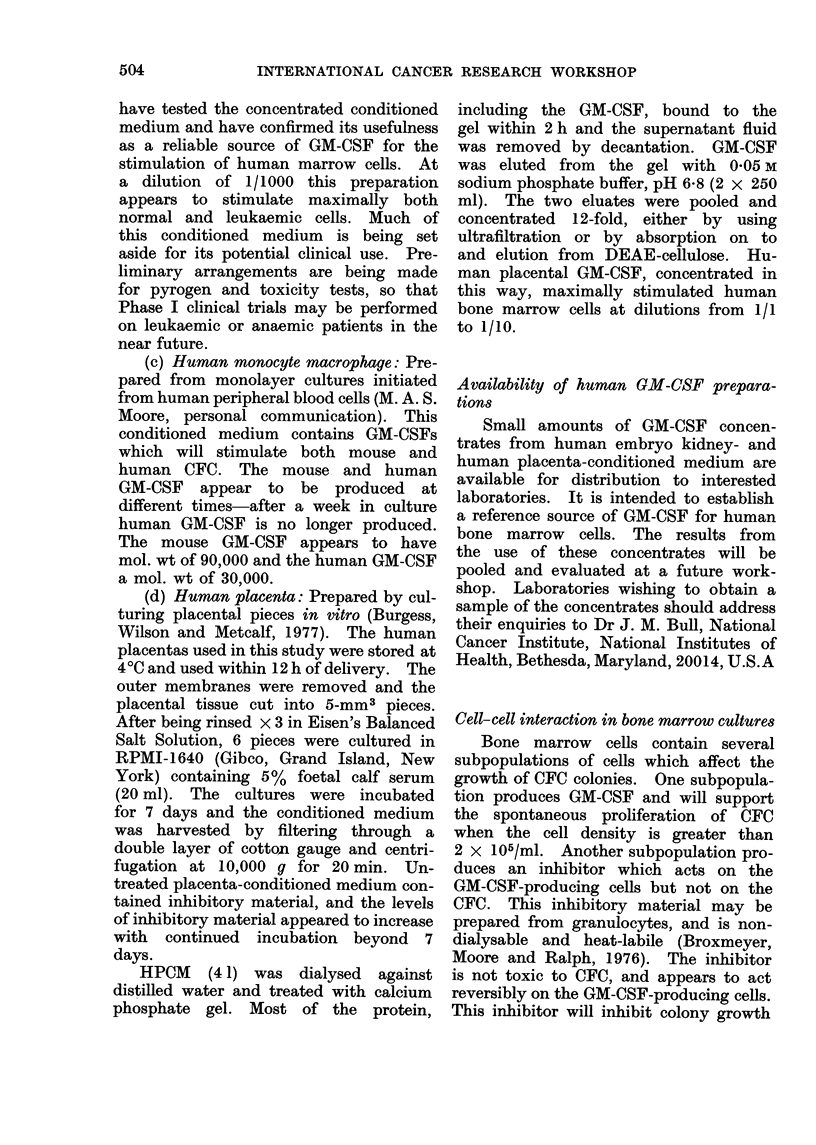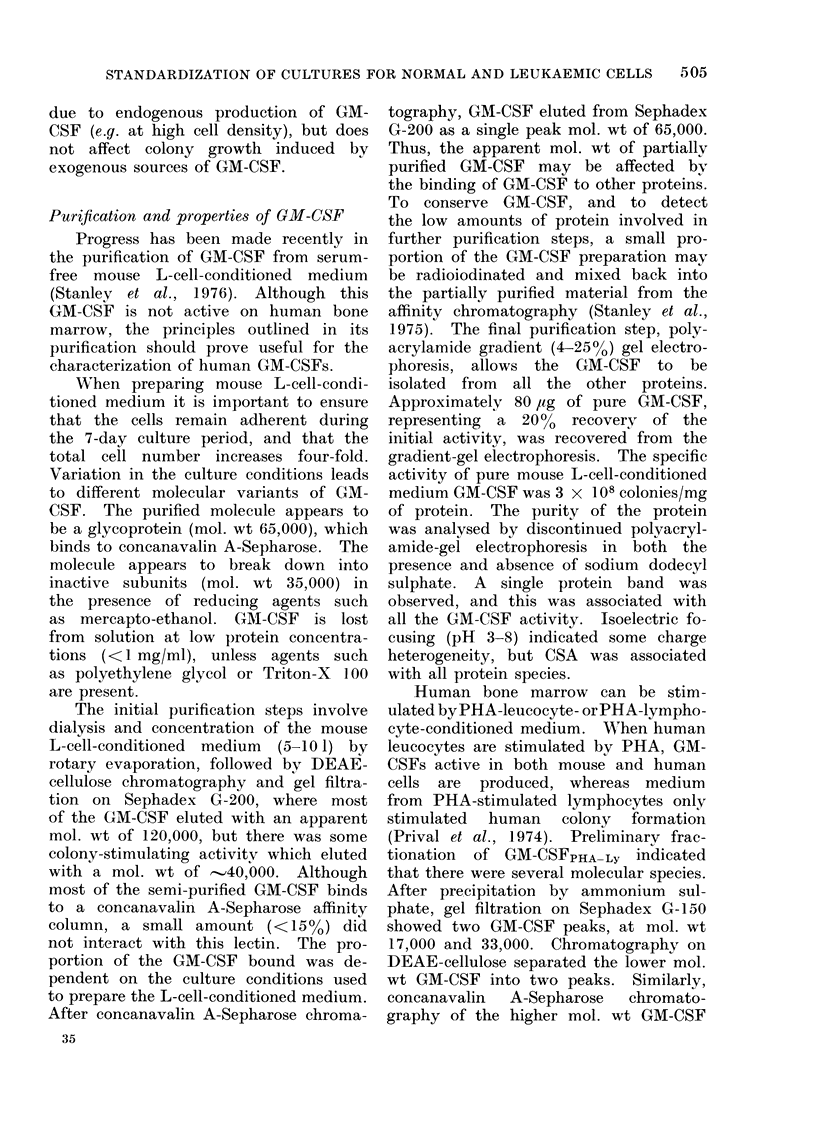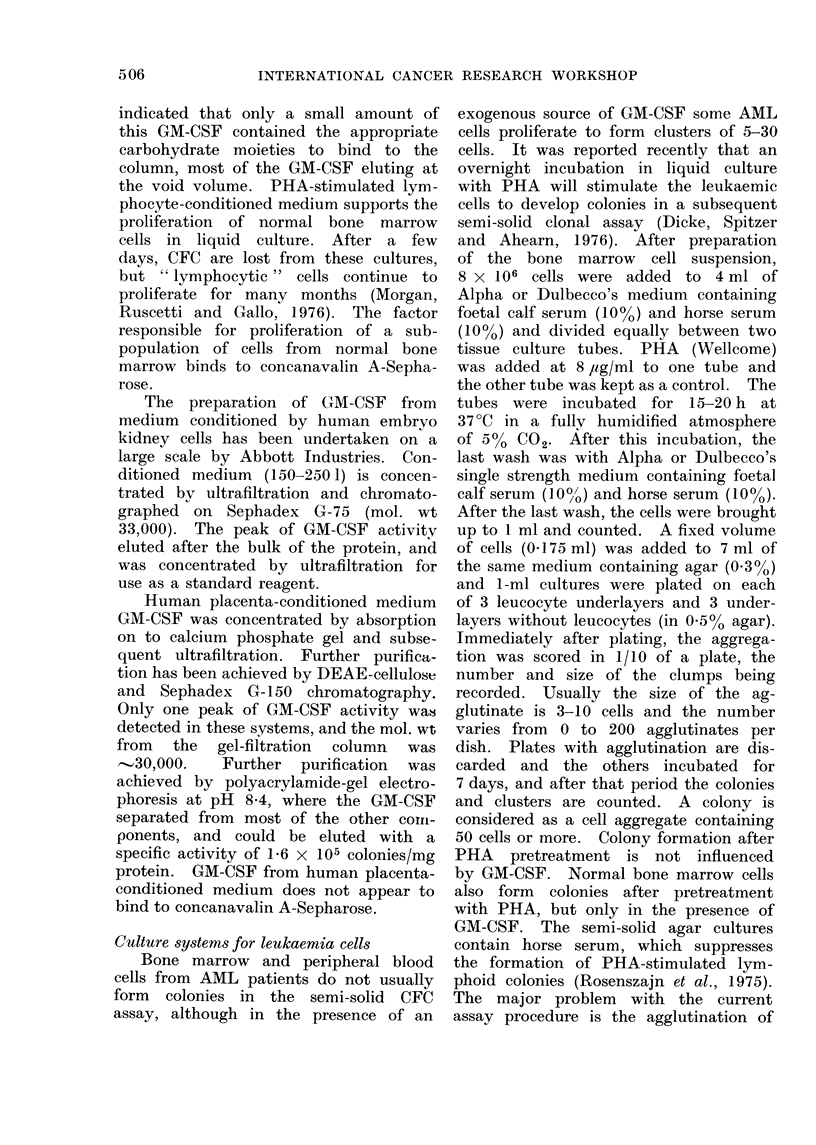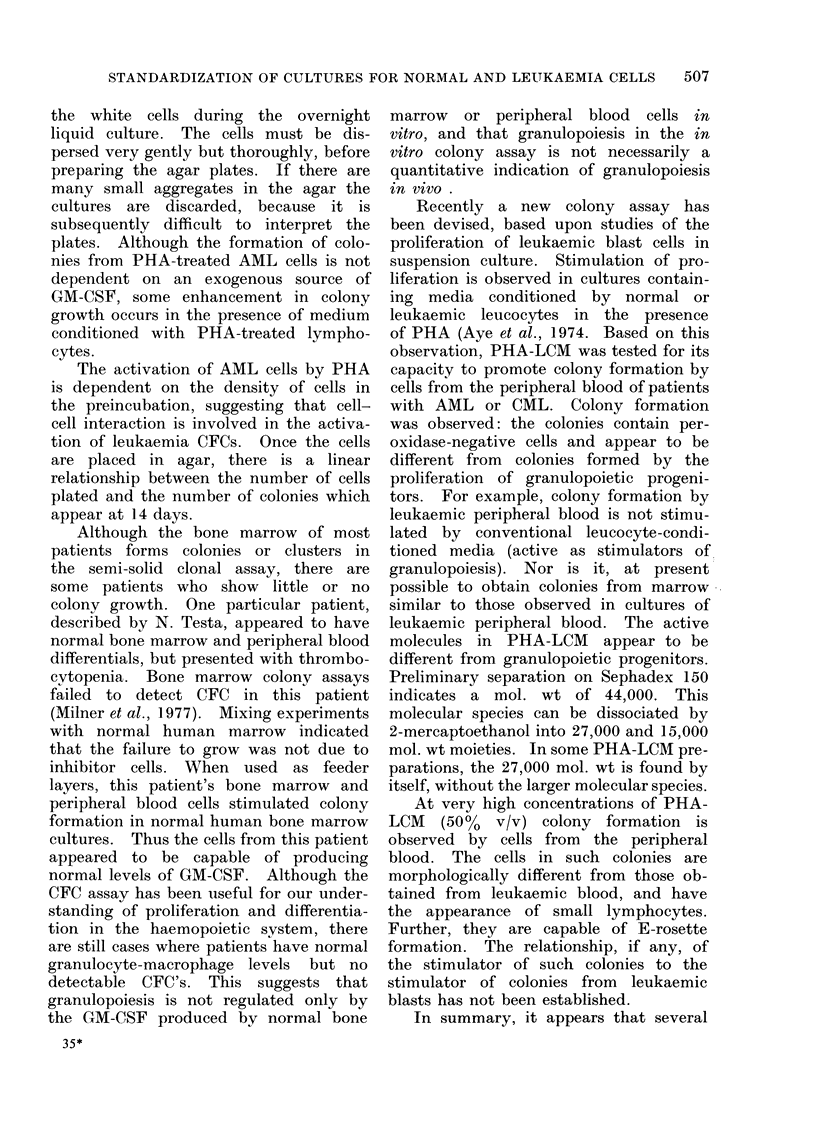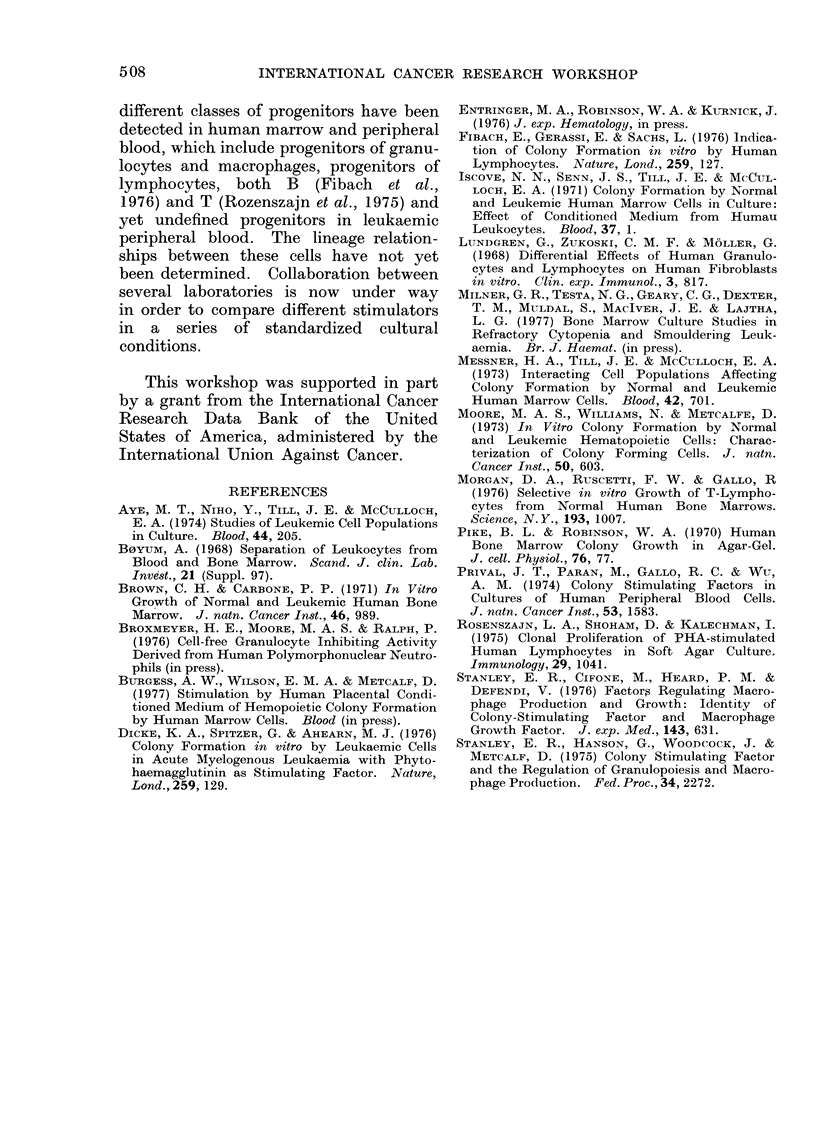# Report of a workshop on standardization of selective cultures for normal and leukaemic cells.

**DOI:** 10.1038/bjc.1977.76

**Published:** 1977-04

**Authors:** 7. A. Moore, A. W. Burgess, D. Metcalf, E. A. McCulloch, W. A. Robinson, K. A. Dicke, P. A. Chervenick, J. M. Bull, A. M. Wu, E. R. Stanley, J. Goldman, N. Testa


*Br. J. Cancer* (1975) **35**, 500

### REPORT OF A WORKSHOP ON STANDARDIZATION OF SELECTIVE CULTURES FOR NORMAL AND LEUKAEMIC CELLS

INTERNATIONAL CANCER RESEARCH WORKSHOP PROGRAMME (ICREW)

**WORKSHOP MEMBERS: CHAIRMAN:** M. A. S. MOORE (New York; **RAPPORTEUR:** A. W. BURGESS (Melbourne); D. METCALF (Melbourne), E. A. McCULLOCH (Toronto), W. A. ROBINSON (Denver), K. A. DICKE (Houston), P. A. CHERVENICK (Pittsburgh), J. M. BULL (Washington), A. M. WU (Washington), E. R. STANLEY (Toronto), J. GOLDMAN (London) and N. TESTA (Manchester)

Received 12 November 1976 Accepted 15 November 1976

*In vitro* cultures of human bone marrow have been used to study normal haemopoiesis and to gain information to aid in the treatment of leukaemia patients. A workshop on the development of semi-solid bone marrow cultures was held for two days in Washington on 15 and 16 August 1976. The participants of this workshop discussed some of the technical problems associated with semi-solid cultures of human marrow, and described methods for obtaining reliable sources of exogenous granulocyte macrophage colony-stimulating factor (GM-CSF)* for human cells. The following report was compiled by the rapporteur from all the oral presentations made at the workshop.

### *Bone marrow collection, preparation and standardization*

There are many critical procedures which must be evaluated properly if bone marrow colony assays are to be used quantitatively. Human bone marrow specimens are invariably diluted to a varying degree with peripheral blood cells and no satisfactory method has been devised to estimate the extent of this contamination. Since peripheral blood contains few colony-forming cells this tends to produce variable frequencies of colony-forming cells (CFC)* in marrow cultures. To minimize the amount of peripheral blood, only cells from the first part of the marrow specimen should be used in the assay. When small bone marrow samples are taken (*e.g.* 0·3 ml) the cellularity of the specimen can increase 2–3-fold, while the number of CFC/$10^5$ nucleated cells appears to remain constant. When using the semi-solid culture system, both the marrow and peripheral blood underlayer cells should be washed free of heparin and autologous plasma. The number of colonies grown using this system with and without autologous plasma is shown in Table I. There is a linear relationship between the number of nucleated cells plated and the number of colonies observed, provided that the number of cells is less than $10^5$/ml. If the cells are cultured at a higher density, colonies are confluent and it is difficult to avoid reaggregation of cultured cells to form pseudo-colonies. The optimal concentration of peripheral blood leucocytes in feeder layers is

Correspondence to Dr A. W. Burgess, Walter and Eliza Hall Institute of Medical Research, P.O. Royal Melbourne Hospital, Parkville, Victoria, Australia, 3050.

* Several alternative abbreviations have been used to represent the progenitors of granulocyte and macrophage colonies. There are differences in philosophy behind the various alternatives and no progress was made towards the adoption of a uniform terminology. An attempt has been made to use a consistent terminology throughout this report, but alternative names are widely used for CFC: *in vitro* CFC and CFU-C. Similarly, granulocyte macrophage colony-stimulating factor (GM-CSF) is also referred to in the literature as colony stimulating activity (CSA) and macrophage/granulocyte inducing factor (MGI).

TABLE I.—*Colonies Grown from Normal Human Marrow Cells when Cultured Over Washed and Unwashed Peripheral Blood Leucocytes (Data of Robinson, W. A., Etringer, M. and Bolin, W. A., University of Colorado)*

| Treatment of underlayer* | Number of marrows† tested | Range of CFC/$10^5$ cells | Mean CFC/$10^5$ cells |
|---|---|---|---|
| None | 99 | 12–120 | 38 |
| Washing | 32 | 20–165 | 69 |

* Underlayers contained $10^6$ cells.

† 2 × $10^5$ marrow cells were cultured for 14 days and aggregates of >40 cells scored as colonies.

$10^6$/ml, and better results are obtained if the polymorphs are first removed from the underlayer cells by density centrifugation over 1·070 g/cm$^3$ bovine serum albumin. Despite the differences in culture techniques there was general agreement that normal bone marrow cells contain 30–50 CFCs/$10^5$ cells. Several groups reported that, although the frequency ranged from 20 to 120 CFCs/$10^5$ on any particular patient, the mean frequencies all fell within the narrow range given above.

Although many cultures are performed with whole washed bone marrow cells, it is often advantageous to fractionate the cells before culture, *e.g.* if it is necessary to culture the cells (a) free of colony-stimulating cells or (b) free of polymorphs, which appear to inhibit colony-stimulating cells. The two most common procedures for bone marrow cell fractionation rely on cell density (to remove polymorphs and some colony-stimulating cells) (Moore, Williams and Metcalf, 1973; Bøyum, 1968) and adherence properties (Messner, Till and McCulloch, 1973; Lundgren, Zukoski and Möller, 1968) to remove colony-stimulating cells. The techniques for cell fractionation do not appear to be routine at present and many technical differences exist between separate laboratories (*e.g.* washing buffer, osmolarity of solutions or speed of centrifugation). Consequently it is important to document both the procedures used and the overall recovery of cells from these procedures. When normal marrow was fractionated by either of these techniques, the recovery of light-density or non-adherent nucleated cells was approximately 50% of the initial cell number and the enrichment of CFCs was 1·5- to 2-fold.

At present there is no standard source of CFCs available for the calibration of the colony assay system. Although bone marrow cells may be stored frozen, the recovery of CFCs on thawing appears to be extremely variable (10–60%). Thus it does not appear to be feasible to distribute a standard source of bone marrow cells. When reporting results for human bone marrow assays, any technical details which might help other laboratories to repeat the published results should be explained in the materials and methods section of the report.

*Semi-solid colony-forming assay*

Several types of bone marrow colony-forming assay are being used currently. In many laboratories double layer cultures are used, where peripheral blood leucocytes are in an under-layer, to stimulate bone marrow cells in a semi-solid over-layer (Pike and Robinson, 1970; Entringer, Robinson and Kurnick, in press). Other laboratories use a single semi-solid layer containing both the bone marrow cells and an exogenous GM-CSF stimulus (Iscove *et al.*, 1971; Prival *et al.*, 1974). Two supporting media (agar and methyl cellulose) are used routinely. The results in both systems depend on the quality of all the reagents used in the culture system, but in general the results are comparable in the two assay systems. Methyl cellulose may be preferable when the experiment requires the harvesting of colony cells, but the agar culture system is essential when assessment of cluster as well as colony numbers is required (*e.g.* AML).

All the components of the CFC assay system must be prepared carefully, and checked to see that they support colony growth. In particular it may be necessary to screen several batches of foetal calf serum before a batch supporting acceptable levels of colony growth is obtained. When using methyl cellulose as the supporting medium, care should be taken in dissolving the powder: the semi-solid medium should be clear and agranular. Incubators must control the pH (~7·2), humidity and temperature (37°C) accurately. The drying out of the semisolid medium during the 7–14-day cultures is a common problem in the CFC assay system. This can be detected by comparing the depth of the agar layer with a freshly prepared plate. Drying out can be minimized by checking that the incubator is fully humidified. When using methyl cellulose it is often necessary to enclose 35-mm Petri dishes inside a larger dish (60-mm) to avoid drying out.

Reagents may be prepared sterile or be rendered sterile by filtration. If the latter method is used, it should be noted that cellulose acetate filters sometimes contain detergent which is inhibitory to colony growth.

*Quantitation of CFCs*

Colony or cluster formation may be scored between 7 and 14 days and, although there is no agreement as to the number of cells which constitute colonies or clusters, it is usually accepted that colonies contain at least 20 cells, although many laboratories only count colonies with more than 40 cells. The results of the CFC assay *are* dependent on the definition of colonies and clusters and the day on which the colonies are scored. Exact methods of scoring should always be stipulated in any publication. The number of human colonies may be maximal at Day 10, and colonies appearing after this time are not necessarily generated from the same population of precursor cells which generated the initial colonies. When colonies are scored for the morphology of the component cells (eosinophil, neutrophil or macrophage), it is apparent that the relative frequency of eosinophil colonies increases with the age of the culture (Table II). These considerations can be important when analysing results from density-gradient or sedimentation-velocity analysis of CFC populations.

TABLE II.—*Colony* (>50 *Cells*) *and Cluster* (5–49 *Cells*) *Morphology in Normal Human Bone Marrow Cultures* ($10^5$ *Cells*) *Stimulated with GM-CSF from Human Placenta-conditioned Medium* (*Data of Dresch, C. and Metcalf, D., Walter and Eliza Hall Institute of Medical Research, Melbourne*)

| | Colonies/$10^5$ cells | | Clusters/$10^5$ cells | |
|---|---|---|---|---|
| Day of scoring | Eosinophil | Neutrophil monocyte | Eosinophil | Neutrophil monocyte |
| 7 | 0·4 | 16 | 61 | 155 |
| 10 | 3 | 27 | 67 | 126 |
| 14 | 11 | 33 | 56 | 107 |

It is essential when attempting to measure CFC levels in cell populations that the GM-CSF concentration is high enough to stimulate all the CFCs present. Many conditioned media used in the past have not delivered a maximal stimulus, although good peripheral blood underlayers seem capable of maximal stimulation. Human embryo kidney and human placental GM-CSF concentrates have been prepared which appear to stimulate maximal colony growth over a 10-fold concentration range. Although the maximal number of colonies stimulated with these conditioned media appears to be slightly higher than peripheral blood underlayers, the colony size is not as large. When suboptimal concentrations of GM-CSF are used, it is difficult to obtain results which are dependent only on the CFC levels in the sample.

*Quantitation of GM-CSF*

When measuring GM-CSF concentrations, care should be taken to subtract background colonies (especially when more than $10^5$ bone marrow cells are plated) and to titrate the sample, in case inhibitors are present. Three laboratories have titrated an identical sample of human embryo kidney GM-CSF, and similar titration curves were found in each of the laboratories, even though the absolute numbers of colonies differed widely (Table III). Many serum and urine samples contain inhibitors, and it is difficult to obtain accurate levels of GM-CSF unless these are actually removed by precipitation or chromatography.

TABLE III.—*Titration of Human Embryo Kidney GM-CSF Concentrate (Abbott Pty Ltd, North Chicago) on* $2 \times 10^5$ *Non-Adherent Human Bone Marrow Cells. Colonies (>20 cells) were Scored Between* 10 *and* 14 *Days*

| Dilution of GM-CSF | Number of colonies/$10^5$ cells | | |
|---|---|---|---|
| | Toronto* | Pittsburgh† | Bethesda‡ |
| 1/200 | 2 | 35 | 10 |
| 1/1000 | 25 | 61 | 42 |
| 1/2000 | — | 47 | 20 |
| 1/3000 | 12 | — | — |

* E. A. McCulloch, Ontario Cancer Institute.
† P. A. Chervenick, University of Pittsburgh.
‡ J. M. Bull, National Cancer Institute.

*Sources of GM-CSF*

(1) *Feederlayers*. — Peripheral blood leucocytes from monkeys or humans may be used as underlayers to stimulate colony growth from human CFC (Pike and Robinson, 1970). Leucocyte underlayers provide excellent stimulation of CFC, but are not reliable in a quantitative sense. Often the underlayers are inactive and the level of stimulation is dependent on the method of storing the underlayers. When leucocyte underlayers are used to provide the exogenous source of CSF more than one batch of underlayers should be used, and the results of the most active underlayer used. Although underlayers may be stored at 37°C in a fully humidified atmosphere of 10% $CO_2$ in air for almost 2 weeks before use in a CFC assay, after the first week the incidence of inactive underlayers increases.

Attempts have been made to prepare conditioned medium which will stimulate growth from human bone marrow cells. Conditioned medium from peripheral blood leucocytes, phytohaemagglutinin (PHA)-stimulated lymphocytes, human embryo kidneys, human monocyte macrophages, human placentas and monkey lungs will all induce colony formation from human bone marrow, but none of these sources will stimulate maximal numbers of colonies unless they are concentrated. In general, the concentrated conditioned media stimulate a similar number of colonies to peripheral blood underlayers, but the size of the colonies is invariably smaller with conditioned media.

(2) *Conditioned media.*—(a) *Peripheral blood leucocytes:* Prepared directly from human peripheral blood leucocytes (Iscove *et al.*, 1971) or by using PHA to stimulate the leucocytes or lymphocytes (Prival *et al.*, 1974).

(b) *Human embryo kidney:* Prepared by Abbott Pty Ltd, North Chicago, U.S.A., under contract to NCI, from medium conditioned by human embryo kidney slices (Brown and Carbone, 1971). This conditioned medium has been concentrated and partially purified by gel-filtration chromatography. Human kidney cell tissue culture harvests (160 l) were treated with an ion exchange resin (to remove urokinase) and concentrated at pH 8·0 by ultrafiltration over a UM-10 membrane (3·2 $ft^2$ surface area) to a protein concentration of 200 mg/ml. After clarification by centrifugation, 400 ml of the concentrate was fractionated on Sephadex G-75 (20 × 120 cm) using tris-HCl (0·01 M, pH 7·4), EDTA (1 mM) and trichlorobutanol (0·1%) as the eluting buffer. The protein eluting between 29 l and 33 l was concentrated by ultrafiltration on a UM-2 membrane to a protein concentration of 250–350 mg/ml and stored frozen. Several laboratories have tested the concentrated conditioned medium and have confirmed its usefulness as a reliable source of GM-CSF for the stimulation of human marrow cells. At a dilution of 1/1000 this preparation appears to stimulate maximally both normal and leukaemic cells. Much of this conditioned medium is being set aside for its potential clinical use. Preliminary arrangements are being made for pyrogen and toxicity tests, so that Phase I clinical trials may be performed on leukaemic or anaemic patients in the near future.

(c) *Human monocyte macrophage:* Prepared from monolayer cultures initiated from human peripheral blood cells (M. A. S. Moore, personal communication). This conditioned medium contains GM-CSFs which will stimulate both mouse and human CFC. The mouse and human GM-CSF appear to be produced at different times—after a week in culture human GM-CSF is no longer produced. The mouse GM-CSF appears to have mol. wt of 90,000 and the human GM-CSF a mol. wt of 30,000.

(d) *Human placenta:* Prepared by culturing placental pieces *in vitro* (Burgess, Wilson and Metcalf, 1977). The human placentas used in this study were stored at 4 °C and used within 12 h of delivery. The outer membranes were removed and the placental tissue cut into 5-$mm^3$ pieces. After being rinsed × 3 in Eisen's Balanced Salt Solution, 6 pieces were cultured in RPMI-1640 (Gibco, Grand Island, New York) containing 5% foetal calf serum (20 ml). The cultures were incubated for 7 days and the conditioned medium was harvested by filtering through a double layer of cotton gauge and centrifugation at 10,000 *g* for 20 min. Untreated placenta-conditioned medium contained inhibitory material, and the levels of inhibitory material appeared to increase with continued incubation beyond 7 days.

HPCM (4 l) was dialysed against distilled water and treated with calcium phosphate gel. Most of the protein, including the GM-CSF, bound to the gel within 2 h and the supernatant fluid was removed by decantation. GM-CSF was eluted from the gel with 0·05 M sodium phosphate buffer, pH 6·8 (2 × 250 ml). The two eluates were pooled and concentrated 12-fold, either by using ultrafiltration or by absorption on to and elution from DEAE-cellulose. Human placental GM-CSF, concentrated in this way, maximally stimulated human bone marrow cells at dilutions from 1/1 to 1/10.

*Availability of human GM-CSF preparations*

Small amounts of GM-CSF concentrates from human embryo kidney- and human placenta-conditioned medium are available for distribution to interested laboratories. It is intended to establish a reference source of GM-CSF for human bone marrow cells. The results from the use of these concentrates will be pooled and evaluated at a future workshop. Laboratories wishing to obtain a sample of the concentrates should address their enquiries to Dr J. M. Bull, National Cancer Institute, National Institutes of Health, Bethesda, Maryland, 20014, U.S.A

*Cell–cell interaction in bone marrow cultures*

Bone marrow cells contain several subpopulations of cells which affect the growth of CFC colonies. One subpopulation produces GM-CSF and will support the spontaneous proliferation of CFC when the cell density is greater than 2 × $10^5$/ml. Another subpopulation produces an inhibitor which acts on the GM-CSF-producing cells but not on the CFC. This inhibitory material may be prepared from granulocytes, and is non-dialysable and heat-labile (Broxmeyer, Moore and Ralph, 1976). The inhibitor is not toxic to CFC, and appears to act reversibly on the GM-CSF-producing cells. This inhibitor will inhibit colony growth due to endogenous production of GM-CSF (*e.g.* at high cell density), but does not affect colony growth induced by exogenous sources of GM-CSF.

*Purification and properties of GM-CSF*

Progress has been made recently in the purification of GM-CSF from serum-free mouse L-cell-conditioned medium (Stanley *et al.*, 1976). Although this GM-CSF is not active on human bone marrow, the principles outlined in its purification should prove useful for the characterization of human GM-CSFs.

When preparing mouse L-cell-conditioned medium it is important to ensure that the cells remain adherent during the 7-day culture period, and that the total cell number increases four-fold. Variation in the culture conditions leads to different molecular variants of GM-CSF. The purified molecule appears to be a glycoprotein (mol. wt 65,000), which binds to concanavalin A-Sepharose. The molecule appears to break down into inactive subunits (mol. wt 35,000) in the presence of reducing agents such as mercapto-ethanol. GM-CSF is lost from solution at low protein concentrations (<1 mg/ml), unless agents such as polyethylene glycol or Triton-X 100 are present.

The initial purification steps involve dialysis and concentration of the mouse L-cell-conditioned medium (5–10 l) by rotary evaporation, followed by DEAE-cellulose chromatography and gel filtration on Sephadex G-200, where most of the GM-CSF eluted with an apparent mol. wt of 120,000, but there was some colony-stimulating activity which eluted with a mol. wt of ~40,000. Although most of the semi-purified GM-CSF binds to a concanavalin A-Sepharose affinity column, a small amount (<15%) did not interact with this lectin. The proportion of the GM-CSF bound was dependent on the culture conditions used to prepare the L-cell-conditioned medium. After concanavalin A-Sepharose chromatography, GM-CSF eluted from Sephadex G-200 as a single peak mol. wt of 65,000. Thus, the apparent mol. wt of partially purified GM-CSF may be affected by the binding of GM-CSF to other proteins. To conserve GM-CSF, and to detect the low amounts of protein involved in further purification steps, a small proportion of the GM-CSF preparation may be radioiodinated and mixed back into the partially purified material from the affinity chromatography (Stanley *et al.*, 1975). The final purification step, polyacrylamide gradient (4–25%) gel electrophoresis, allows the GM-CSF to be isolated from all the other proteins. Approximately 80 μg of pure GM-CSF, representing a 20% recovery of the initial activity, was recovered from the gradient-gel electrophoresis. The specific activity of pure mouse L-cell-conditioned medium GM-CSF was $3 \times 10^8$ colonies/mg of protein. The purity of the protein was analysed by discontinued polyacrylamide-gel electrophoresis in both the presence and absence of sodium dodecyl sulphate. A single protein band was observed, and this was associated with all the GM-CSF activity. Isoelectric focusing (pH 3–8) indicated some charge heterogeneity, but CSA was associated with all protein species.

Human bone marrow can be stimulated by PHA-leucocyte- or PHA-lymphocyte-conditioned medium. When human leucocytes are stimulated by PHA, GM-CSFs active in both mouse and human cells are produced, whereas medium from PHA-stimulated lymphocytes only stimulated human colony formation (Prival *et al.*, 1974). Preliminary fractionation of $\text{GM-CSF}_{\text{PHA-Ly}}$ indicated that there were several molecular species. After precipitation by ammonium sulphate, gel filtration on Sephadex G-150 showed two GM-CSF peaks, at mol. wt 17,000 and 33,000. Chromatography on DEAE-cellulose separated the lower mol. wt GM-CSF into two peaks. Similarly, concanavalin A-Sepharose chromatography of the higher mol. wt GM-CSF

indicated that only a small amount of this GM-CSF contained the appropriate carbohydrate moieties to bind to the column, most of the GM-CSF eluting at the void volume. PHA-stimulated lymphocyte-conditioned medium supports the proliferation of normal bone marrow cells in liquid culture. After a few days, CFC are lost from these cultures, but " lymphocytic " cells continue to proliferate for many months (Morgan, Ruscetti and Gallo, 1976). The factor responsible for proliferation of a subpopulation of cells from normal bone marrow binds to concanavalin A-Sepharose.

The preparation of GM-CSF from medium conditioned by human embryo kidney cells has been undertaken on a large scale by Abbott Industries. Conditioned medium (150–250 l) is concentrated by ultrafiltration and chromatographed on Sephadex G-75 (mol. wt 33,000). The peak of GM-CSF activity eluted after the bulk of the protein, and was concentrated by ultrafiltration for use as a standard reagent.

Human placenta-conditioned medium GM-CSF was concentrated by absorption on to calcium phosphate gel and subsequent ultrafiltration. Further purification has been achieved by DEAE-cellulose and Sephadex G-150 chromatography. Only one peak of GM-CSF activity was detected in these systems, and the mol. wt from the gel-filtration column was ~30,000. Further purification was achieved by polyacrylamide-gel electrophoresis at pH 8·4, where the GM-CSF separated from most of the other components, and could be eluted with a specific activity of $1{\cdot}6 \times 10^5$ colonies/mg protein. GM-CSF from human placenta-conditioned medium does not appear to bind to concanavalin A-Sepharose.

*Culture systems for leukaemia cells*

Bone marrow and peripheral blood cells from AML patients do not usually form colonies in the semi-solid CFC assay, although in the presence of an exogenous source of GM-CSF some AML cells proliferate to form clusters of 5–30 cells. It was reported recently that an overnight incubation in liquid culture with PHA will stimulate the leukaemic cells to develop colonies in a subsequent semi-solid clonal assay (Dicke, Spitzer and Ahearn, 1976). After preparation of the bone marrow cell suspension, $8 \times 10^6$ cells were added to 4 ml of Alpha or Dulbecco's medium containing foetal calf serum (10%) and horse serum (10%) and divided equally between two tissue culture tubes. PHA (Wellcome) was added at 8 μg/ml to one tube and the other tube was kept as a control. The tubes were incubated for 15–20 h at 37 °C in a fully humidified atmosphere of 5% $CO_2$. After this incubation, the last wash was with Alpha or Dulbecco's single strength medium containing foetal calf serum (10%) and horse serum (10%). After the last wash, the cells were brought up to 1 ml and counted. A fixed volume of cells (0·175 ml) was added to 7 ml of the same medium containing agar (0·3%) and 1-ml cultures were plated on each of 3 leucocyte underlayers and 3 underlayers without leucocytes (in 0·5% agar). Immediately after plating, the aggregation was scored in 1/10 of a plate, the number and size of the clumps being recorded. Usually the size of the agglutinate is 3–10 cells and the number varies from 0 to 200 agglutinates per dish. Plates with agglutination are discarded and the others incubated for 7 days, and after that period the colonies and clusters are counted. A colony is considered as a cell aggregate containing 50 cells or more. Colony formation after PHA pretreatment is not influenced by GM-CSF. Normal bone marrow cells also form colonies after pretreatment with PHA, but only in the presence of GM-CSF. The semi-solid agar cultures contain horse serum, which suppresses the formation of PHA-stimulated lymphoid colonies (Rosenszajn *et al.*, 1975). The major problem with the current assay procedure is the agglutination of the white cells during the overnight liquid culture. The cells must be dispersed very gently but thoroughly, before preparing the agar plates. If there are many small aggregates in the agar the cultures are discarded, because it is subsequently difficult to interpret the plates. Although the formation of colonies from PHA-treated AML cells is not dependent on an exogenous source of GM-CSF, some enhancement in colony growth occurs in the presence of medium conditioned with PHA-treated lymphocytes.

The activation of AML cells by PHA is dependent on the density of cells in the preincubation, suggesting that cell–cell interaction is involved in the activation of leukaemia CFCs. Once the cells are placed in agar, there is a linear relationship between the number of cells plated and the number of colonies which appear at 14 days.

Although the bone marrow of most patients forms colonies or clusters in the semi-solid clonal assay, there are some patients who show little or no colony growth. One particular patient, described by N. Testa, appeared to have normal bone marrow and peripheral blood differentials, but presented with thrombocytopenia. Bone marrow colony assays failed to detect CFC in this patient (Milner *et al.*, 1977). Mixing experiments with normal human marrow indicated that the failure to grow was not due to inhibitor cells. When used as feeder layers, this patient's bone marrow and peripheral blood cells stimulated colony formation in normal human bone marrow cultures. Thus the cells from this patient appeared to be capable of producing normal levels of GM-CSF. Although the CFC assay has been useful for our understanding of proliferation and differentiation in the haemopoietic system, there are still cases where patients have normal granulocyte-macrophage levels but no detectable CFC's. This suggests that granulopoiesis is not regulated only by the GM-CSF produced by normal bone marrow or peripheral blood cells *in vitro*, and that granulopoiesis in the *in vitro* colony assay is not necessarily a quantitative indication of granulopoiesis *in vivo* .

Recently a new colony assay has been devised, based upon studies of the proliferation of leukaemic blast cells in suspension culture. Stimulation of proliferation is observed in cultures containing media conditioned by normal or leukaemic leucocytes in the presence of PHA (Aye *et al.*, 1974. Based on this observation, PHA-LCM was tested for its capacity to promote colony formation by cells from the peripheral blood of patients with AML or CML. Colony formation was observed: the colonies contain peroxidase-negative cells and appear to be different from colonies formed by the proliferation of granulopoietic progenitors. For example, colony formation by leukaemic peripheral blood is not stimulated by conventional leucocyte-conditioned media (active as stimulators of granulopoiesis). Nor is it, at present possible to obtain colonies from marrow similar to those observed in cultures of leukaemic peripheral blood. The active molecules in PHA-LCM appear to be different from granulopoietic progenitors. Preliminary separation on Sephadex 150 indicates a mol. wt of 44,000. This molecular species can be dissociated by 2-mercaptoethanol into 27,000 and 15,000 mol. wt moieties. In some PHA-LCM preparations, the 27,000 mol. wt is found by itself, without the larger molecular species.

At very high concentrations of PHA-LCM (50% v/v) colony formation is observed by cells from the peripheral blood. The cells in such colonies are morphologically different from those obtained from leukaemic blood, and have the appearance of small lymphocytes. Further, they are capable of E-rosette formation. The relationship, if any, of the stimulator of such colonies to the stimulator of colonies from leukaemic blasts has not been established.

In summary, it appears that several different classes of progenitors have been detected in human marrow and peripheral blood, which include progenitors of granulocytes and macrophages, progenitors of lymphocytes, both B (Fibach *et al.*, 1976) and T (Rozenszajn *et al.*, 1975) and yet undefined progenitors in leukaemic peripheral blood. The lineage relationships between these cells have not yet been determined. Collaboration between several laboratories is now under way in order to compare different stimulators in a series of standardized cultural conditions.

This workshop was supported in part by a grant from the International Cancer Research Data Bank of the United States of America, administered by the International Union Against Cancer.

### REFERENCES

AYE, M. T., NIHO, Y., TILL, J. E. & MCCULLOCH, E. A. (1974) Studies of Leukemic Cell Populations in Culture. *Blood*, **44**, 205.

BØYUM, A. (1968) Separation of Leukocytes from Blood and Bone Marrow. *Scand. J. clin. Lab. Invest.*, **21** (Suppl. 97).

BROWN, C. H. & CARBONE, P. P. (1971) *In Vitro* Growth of Normal and Leukemic Human Bone Marrow. *J. natn. Cancer Inst.*, **46**, 989.

BROXMEYER, H. E., MOORE, M. A. S. & RALPH, P. (1976) Cell-free Granulocyte Inhibiting Activity Derived from Human Polymorphonuclear Neutrophils (in press).

BURGESS, A. W., WILSON, E. M. A. & METCALF, D. (1977) Stimulation by Human Placental Conditioned Medium of Hemopoietic Colony Formation by Human Marrow Cells. *Blood* (in press).

DICKE, K. A., SPITZER, G. & AHEARN, M. J. (1976) Colony Formation *in vitro* by Leukaemic Cells in Acute Myelogenous Leukaemia with Phytohaemagglutinin as Stimulating Factor. *Nature, Lond.*, **259**, 129.

ENTRINGER, M. A., ROBINSON, W. A. & KURNICK, J. (1976) *J. exp. Hematology*, in press.

FIBACH, E., GERASSI, E. & SACHS, L. (1976) Indication of Colony Formation *in vitro* by Human Lymphocytes. *Nature, Lond.*, **259**, 127.

ISCOVE, N. N., SENN, J. S., TILL, J. E. & MCCULLOCH, E. A. (1971) Colony Formation by Normal and Leukemic Human Marrow Cells in Culture: Effect of Conditioned Medium from Humau Leukocytes. *Blood*, **37**, 1.

LUNDGREN, G., ZUKOSKI, C. M. F. & MÖLLER, G. (1968) Differential Effects of Human Granulocytes and Lymphocytes on Human Fibroblasts *in vitro*. *Clin. exp. Immunol.*, **3**, 817.

MILNER, G. R., TESTA, N. G., GEARY, C. G., DEXTER, T. M., MULDAL, S., MACIVER, J. E. & LAJTHA, L. G. (1977) Bone Marrow Culture Studies in Refractory Cytopenia and Smouldering Leukaemia. *Br. J. Haemat.* (in press).

MESSNER, H. A., TILL, J. E. & MCCULLOCH, E. A. (1973) Interacting Cell Populations Affecting Colony Formation by Normal and Leukemic Human Marrow Cells. *Blood*, **42**, 701.

MOORE, M. A. S., WILLIAMS, N. & METCALFE, D. (1973) *In Vitro* Colony Formation by Normal and Leukemic Hematopoietic Cells: Characterization of Colony Forming Cells. *J. natn. Cancer Inst.*, **50**, 603.

MORGAN, D. A., RUSCETTI, F. W. & GALLO, R (1976) Selective *in vitro* Growth of T-Lymphocytes from Normal Human Bone Marrows. *Science, N.Y.*, **193**, 1007.

PIKE, B. L. & ROBINSON, W. A. (1970) Human Bone Marrow Colony Growth in Agar-Gel. *J. cell. Physiol.*, **76**, 77.

PRIVAL, J. T., PARAN, M., GALLO, R. C. & WU, A. M. (1974) Colony Stimulating Factors in Cultures of Human Peripheral Blood Cells. *J. natn. Cancer Inst.*, **53**, 1583.

ROSENSZAJN, L. A., SHOHAM, D. & KALECHMAN, I. (1975) Clonal Proliferation of PHA-stimulated Human Lymphocytes in Soft Agar Culture. *Immunology*, **29**, 1041.

STANLEY, E. R., CIFONE, M., HEARD, P. M. & DEFENDI, V. (1976) Factors Regulating Macrophage Production and Growth: Identity of Colony-Stimulating Factor and Macrophage Growth Factor. *J. exp. Med.*, **143**, 631.

STANLEY, E. R., HANSON, G., WOODCOCK, J. & METCALF, D. (1975) Colony Stimulating Factor and the Regulation of Granulopoiesis and Macrophage Production. *Fed. Proc.*, **34**, 2272.